# Understanding face matching

**DOI:** 10.1177/17470218221104476

**Published:** 2022-06-17

**Authors:** Matthew C Fysh, Markus Bindemann

**Affiliations:** School of Psychology, University of Kent, Canterbury, UK

**Keywords:** Unfamiliar face matching, accuracy, similarity, dissociation

## Abstract

Many security settings rely on the identity matching of unfamiliar people, which has led this task to be studied extensively in Cognitive Psychology. In these experiments, observers typically decide whether pairs of faces depict one person (an identity match) or two different people (an identity mismatch). The visual similarity of the to-be-compared faces must play a primary role in how observers accurately resolve this task, but the nature of this similarity–accuracy relationship is unclear. The current study investigated the association between accuracy and facial similarity at the level of individual items (Experiments 1 and 2) and facial features (Experiments 3 and 4). All experiments demonstrate a strong link between similarity and matching accuracy, indicating that this forms the basis of identification decisions. At a feature level, however, similarity exhibited distinct relationships with match and mismatch accuracy. In matches, similarity information was generally shared across the features of a face pair under comparison, with greater similarity linked to higher accuracy. Conversely, features within mismatching face pairs exhibited greater variation in similarity information. This indicates that identity matches and mismatches are characterised by different similarity profiles, which present distinct challenges to the cognitive system. We propose that these identification decisions can be resolved through the accumulation of convergent featural information in matches and the evaluation of divergent featural information in mismatches.

## Introduction

In recent years, the identity matching of unfamiliar faces has been studied extensively in Psychology (see, for example, Bindemann, 2021; Fysh & Bindemann, 2017a; Lander et al., 2018). In this task, observers typically compare unfamiliar faces to determine whether these depict the same person. Due to the importance of face matching for applied settings, such as passport control or person identification in criminal investigations, much research has focused on identifying real-world variables that influence this task, such as time pressure (e.g., Bindemann et al., 2016; Fysh & Bindemann, 2017b; Wirth & Carbon, 2017), expertise (e.g., Bate et al., 2019; Towler et al., 2017; White et al., 2014), and natural variability in facial appearance (e.g., Bindemann & Sandford, 2011; Jenkins et al., 2011; Megreya et al., 2013; Mileva et al., 2020). Consequently, many factors are now understood to impact face-matching performance in applied settings.

In contrast, comparatively little progress has been made towards establishing a theory of *how* observers match the identities of unfamiliar faces. This knowledge gap is important as face identification proceeds along a continuum (see, for example, Burton, 2013; Jenkins & Burton, 2011; Kramer et al., 2018; Young & Burton, 2018). On one end of this continuum lies the recognition of familiar faces, which has been studied for many decades in Psychology (e.g., Benton, 1980; Bruce, 1983; Bruce & Young, 1986; Burton et al., 1990; Cross et al., 1971; Ellis, 1975). Face matching provides an entry point to the opposite end of this continuum, by speaking of how faces can be identified in the absence of familiarity. In addition, face matching provides insight into how the cognitive system supports face identification in the context of emergent technology. For example, whereas the applied task of unfamiliar-face matching is now utilised on a global scale, it still presents a relatively novel challenge for the human cognitive system. Prior to the widespread availability of photography in the 20th century, the modern-day customs of identifying unfamiliar people from photographs did not exist at all (White et al., 2021).

One of the primary obstacles to devising a theory of face matching lies in a key aspect of this task. On seeing a pair of faces, observers must decide whether it constitutes two photographs of the same person (an identity *match*) or of different people (a *mismatch*). Paradoxically, while these types of face pairings seem to present obverse aspects of the same task, their classification appears to be driven by distinct processes. For example, when observers are asked to classify matches and mismatches that are derived from the same face images, by-item analyses of identification decisions reveal no association in accuracy across both types of face pairings (Megreya & Burton, 2007; see also Megreya et al., 2011; Sauerland et al., 2016). This finding is remarkable considering that a strong by-item correlation for matches and mismatches is found when the target identities have been familiarised prior to identification (Megreya & Burton, 2007). Thus, familiarised faces that are easier to recognise as an identity match are also easier to reject as a match to another person, but this match–mismatch association is absent when the same faces are unfamiliar to the observer. The dissociation between match and mismatch decisions also runs counter to an established psychological phenomenon. According to the Mirror Effect, objects that are recognised more accurately as “old” should also be recognised more accurately as “new” (Glanzer & Adams, 1985; Glanzer et al., 1993). This effect has been observed across a range of stimulus categories, such as words or pictures of scenes, animals, and objects (see, for example, Glanzer & Bowles, 1976; Snodgrass et al., 1978; for a review see Glanzer & Adams, 1985). For unfamiliar face matching, this association between positive and negative identifications breaks down, but the reason for this is unclear.

A dissociation between match and mismatch accuracy is also apparent at the *observer* level. While broad individual differences in face-matching ability have been reported (for reviews, see Bate et al., 2021; Lander et al., 2018), a positive association between match and mismatch accuracy, which would suggest that these face pairings are classified in corresponding ways, is not typically found. In contrast, weak negative correlations are sometimes observed. Megreya and Burton (2007), for example, found no consistent relationship between performance on match and mismatch trials across a series of experiments, with weak non-significant correlations that were sometimes trending towards a negative relationship (e.g., *Pearson’s r* = −.11/−.26 for stimulus Sets 1 and 2 of Experiment 1). Other studies have produced similar findings (e.g., Bate et al., 2018, 2019; Kokje et al., 2018), indicating response tendencies towards match or mismatch decisions, though these depend on context. Performance on *mismatch* trials, for example, can deteriorate over prolonged testing sessions, while accuracy on match trials remains largely stable (Alenezi & Bindemann, 2013; Alenezi et al., 2015; Papesh et al., 2018). Similarly, mismatch accuracy is selectively impaired when one face image is embedded within a passport-style frame (McCaffery & Burton, 2016), as well as when matching faces under time pressure (Bindemann et al., 2016; Fysh & Bindemann, 2017b), and when observers are sleep-deprived (Beattie et al., 2016). Conversely, match accuracy appears to suffer to a much greater extent than mismatch accuracy when one face within a pair is visually degraded (Bindemann et al., 2013; Ritchie et al., 2018; Strathie & McNeill, 2016). Studies with face memory tasks have demonstrated comparable findings, by showing that identification accuracy on target-present and target-absent lineups can be manipulated selectively (e.g., Oriet & Fitzgerald, 2018; see also, Bate et al., 2015).

Together, these findings provide converging evidence that identity match and mismatch face pairings are treated as distinct challenges by the cognitive system, and thus require different processes to solve. This could account for why the identification of matches and mismatches is not associated at the *item* level (Megreya & Burton, 2007), and why response biases emerge at the *observer* level (e.g., Alenezi et al., 2015; McCaffery & Burton, 2016). Accordingly, the correct balance between match and mismatch judgements may be difficult to strike, particularly under viewing conditions in which identification decisions are challenging (e.g., Alenezi & Bindemann, 2013; Bindemann et al., 2013; McCaffery & Burton, 2016; Ritchie et al., 2018). This could lead observers to classify a disproportionate percentage of face pairs as matches or mismatches during identification.

However, whereas this theory could explain the dissociation between match and mismatch decisions, it also appears difficult to reconcile with a specific property of unfamiliar-face matching. In this task, observers only have access to the pictorial information contained within face photographs (see, for example, Burton, 2013; Jenkins & Burton, 2011). Face matching is therefore an *image-bound* process, whereby comparisons between identities are constrained by the visual information within a given face photograph (see Fysh & Bindemann, 2017a; Hancock et al., 2000; Jenkins & Burton, 2011). Consequently, both match and mismatch decisions must reflect the visual overlap between the to-be-compared face images at hand, with similar pairings identified as matches and dissimilar pairings as mismatches.

This reasoning receives support from a range of findings. For example, when the similarity of two face identities under comparison is manipulated gradually along a metrically quantifiable continuum, by morphing one face into another, matching accuracy varies accordingly (Robertson, Kramer, et al., 2017). And identity matches that are perceived a priori to exhibit low visual similarity are more likely to be classified as different people than those that receive high-similarity ratings. In turn, high-similarity mismatches are correspondingly more likely to be classed as the same person than low-similarity mismatches (Papesh et al., 2018; Rice et al., 2013). Finally, unfamiliar-face matching also correlates with performance in other visual comparison tasks that require observers to detect similarities or discrepancies between non-face objects (Burton et al., 2010; Megreya & Burton, 2006).

Despite these findings, the specific nature of the relationship between similarity and accuracy is not clear. Some studies have investigated the importance of similarity at the level of individual facial features, providing a breakdown of face regions that may be informative for identity matching (Rice et al., 2013; Towler et al., 2019, 2014, 2017). However, these studies do not distinguish between features that are useful for *match* versus *mismatch* decisions. As a consequence, the extent to which visual similarity relates to both types of decisions, and how this might be reconciled with a theory in which the identification of these stimuli dissociates, remains unknown.

In this study, we investigate how similarity can be reconciled with the match–mismatch dissociation in face matching. We begin by comparing matching accuracy with similarity ratings for pairs of face stimuli. A key issue here is to determine the relationship of similarity and face-matching accuracy *separately* for match and mismatch trials. If both decisions derive from an assessment of the visual similarity between stimuli, then this should be characterised by a positive relationship with accuracy for face matches (i.e., high similarity, high accuracy) and a negative relationship for mismatches (i.e., low similarity, high accuracy). The question of main interest is whether such relationships do, in fact, exist, considering that the identification accuracy of matches does not correlate with that of mismatches.

## Experiment 1

This experiment explored the accuracy–similarity relationship for match and mismatch trials. To contrast these results with the match–mismatch dissociation in face matching, it is important to examine this under conditions in which matches and mismatches are generated from the same identities. In line with previous work, a by-item analysis of these face pairs should then reveal a dissociation in the classification of these trial types (Megreya & Burton, 2007). The question of main interest is whether similarity correlates with the classification of match *and* mismatch trials, even when accuracy for these trial subcomponents does not.

An existing face test was adapted to investigate this, by presenting observers with the mismatch stimuli from the Kent Face Matching Test (KFMT; Fysh & Bindemann, 2018) and two sets of matches that were constructed from the identities of these mismatches. This design therefore yields two opportunities for investigating the match–mismatch dissociation. We then collected similarity ratings for all face pairings to investigate how these map onto the accuracy relationship of matches and mismatches.

Considering that identification decisions should reflect the visual overlap between to-be-compared faces, and based on the indirect evidence that similarity plays an important role in this process (Papesh et al., 2018; Rice et al., 2013; White et al., 2013), positive correlations between accuracy and similarity on *match* trials, and negative correlations between accuracy and similarity on *mismatch* trials, should emerge. However, considering that match and mismatch accuracy does not correlate positively, it was also possible that similarity displays an unexpected relationship with these trial types.

### Method

#### Participants

In all, 71 undergraduates (60 females, 10 males, and 1 undisclosed) from the University of Kent, with a mean age of 19 years (*SD* = 1.00), took part in this experiment for course credit. All experiments reported here were conducted in accordance with the ethical guidelines of the British Psychological Association and approved by the ethics committee of the School of Psychology at the University of Kent.

#### Stimuli

The stimuli consisted of 60 pairs of Caucasian faces (30 females, 30 males) from the KFMT (Fysh & Bindemann, 2018). Each face-pair stimulus comprised one high-quality digital photograph measuring 283 × 332 pixels of a person under controlled lighting with a neutral expression and pose, which was positioned on the right-hand side of a white background. The left-hand image in each pair was a student ID photograph measuring 142 × 192 pixels. These student ID images were not subject to the restrictions placed upon the controlled laboratory photograph, and thus varied regarding lighting, expression, and pose. Both images were presented at a resolution of 72-ppi.

**Figure 1.**
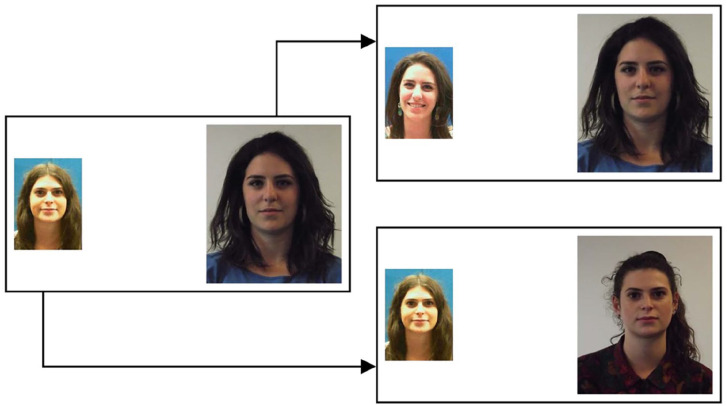
Example stimuli from Experiment 1. The face pair on the left illustrates an identity mismatch. The face pairs on the right depict the two matches that were generated from this mismatch, for Set A (upper) and Set B (lower).

A total of 20 of the 60 face pairs consisted of the identity mismatch pairs that feature in the short version of the KFMT (see Fysh & Bindemann, 2018). The remaining stimuli were 40 matches that were constructed from these mismatched identities. Thus, for every mismatch pairing two matches were generated. These 40 matches were divided into two sets of 20 face pairs. Set A matches were those that were derived from the left image of each mismatch pair, and Set B matches were those that were derived from the right image. An example mismatch pair, with its Set A and Set B match counterpart, is depicted in Figure 1.

#### Procedure

This study was conducted online using *Qualtrics* software. Stimuli were presented one at a time in a random order, along with the question, “Do the above two faces depict the same person or different individuals?” Participants responded as “same” or “different” by clicking on the relevant response. In a second block of trials, observers then viewed each stimulus pair once more in a randomised order and rated the similarity of each pair on a 7-point scale, with options 1 and 7 corresponding to *Not at all similar* and *Highly similar*, respectively.

### Results^1^

#### Accuracy

To investigate the relationship between trial types, the percentage accuracy for mismatch face pairs and the two sets of match pairs was calculated on a by-item basis and correlations were performed. No correlation was found between Set A matches and the mismatches, *r* = −.13, *p* = .591, or between Set B matches and the mismatches, *r* = .34, *p* = .137. A correlation was also absent when these data were combined, *r* = .16, *p* = .334.

We also investigated the match–mismatch relationship on a by-subject basis. Accuracy correlated positively between the two sets of matches, *r* = .68, *p* < .001, whereas a weak negative correlation was observed between Set A matches and the mismatches, *r* = −.28, *p* = .018, and between Set B matches and the mismatches, *r* = −.25, *p* = .033.

#### Accuracy-similarity correlations

Next, the accuracy–similarity relationship was explored on a by-item basis. For this purpose, we first examined whether mean similarity ratings varied for correctly classified matches and mismatches with a one-way ANOVA (Set A matches, Set B matches, mismatches), which revealed an effect of stimulus set, *F*(2,57) = 125.15, *p* < .001, η_*p*_^2^ = .82. Independent sample *t*-tests showed that this was driven by higher similarity ratings for Set A (*M* = 5.67, *SD* = 0.48) and Set B matches (*M* = 5.56, *SD* = 0.71) relative to the mismatches (*M* = 2.97, *SD* = 0.62), *t*(38) = 15.37, *p* < .001, *d* = 4.86 and *t*(38) = 12.28, *p* < .001, *d* = 3.88, respectively. Mean similarity was comparable between Set A and Set B matches, *t*(38) = 0.58, *p* = .569, *d* = 0.18.

**Figure 2.**
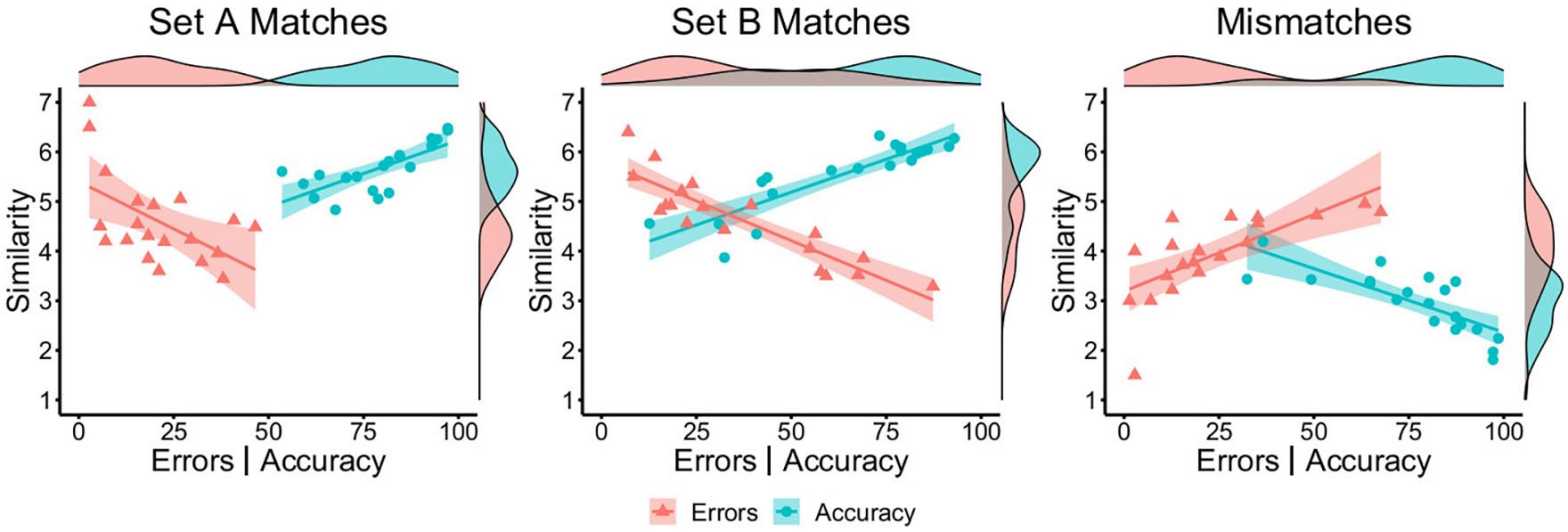
By-item correlations of accuracy (blue circles) and error percentage (red triangles) with similarity ratings for Set A matches (left), Set B matches (middle), and mismatches (right) in Experiment 1.

A series of Pearson’s correlations were next conducted to investigate whether correct and incorrect identity classifications are linked to the perceived similarity of the two faces under comparison. These show that accuracy and similarity scores correlated strongly and positively for Set A matches, *r* = .73, *p* < .001, and for Set B matches, *r* = .88, *p* < .001. Conversely, accuracy and similarity correlated strongly and negatively for mismatches, *r* = −.79, *p* < .001. A complementary pattern of correlations was observed for face pairs that were classified incorrectly, whereby mean similarity scores correlated negatively with error rates for match Set A, *r* = −.55, *p* = .011, and for match Set B, *r* = −.90, *p* < .001, and positively with error rates for mismatches, *r* = .71, *p* < .001. These correlations are illustrated in Figure 2.

#### Perceived similarity

The accuracy–similarity correlations suggest that similarity judgements for pairs of faces followed the same pattern on correct and incorrect trials. To investigate this directly, we compared the similarity ratings for all items when these were classified correctly and incorrectly. First, the similarity ratings were correlated for correct and incorrect face-matching decisions on a by-item basis. To understand how these relationships can be reconciled with response accuracy, the mean similarity ratings for correctly and incorrectly classified matches and mismatches were then compared using independent-samples *t*-tests.

This analysis revealed strong positive correlations of similarity ratings for the matches of Set A, *r* = .78, *p* < .001, and Set B, *r* = .82, *p* < .001, and for mismatches, *r* = .70, *p* < .001. However, correctly classified face pairs received higher similarity ratings than when they were classified incorrectly in match Set A (*M* = 5.67, *SD* = 0.48 vs. *M* = 4.60, *SD* = 0.91), *t*(38) = 4.67, *p* < .001, *d* = 1.48, and match Set B (*M* = 5.56, *SD* = 0.71 vs. *M* = 4.66, *SD* = 0.84), *t*(38) = 3.67, *p* < .001, *d* = 1.16. Conversely, correctly classified mismatches received lower similarity ratings (*M* = 2.97, *SD* = 0.62) than incorrectly classified mismatch pairs (*M* = 3.93, *SD* = 0.83), *t*(38) = 4.11, *p* < .001, *d* = 1.30. This indicates that the perceived similarity of faces varies, depending on whether face pairings are classified correctly or not.

### Discussion

In this experiment, no association between match and mismatch accuracy was found on a by-item basis. And on a by-subject basis, accuracy correlated positively and strongly across the two sets of matches, but both sets of matches correlated negatively and weakly with mismatch accuracy. These findings therefore converge with the match–mismatch dissociation that has been observed in previous studies for the same face identities (e.g., Megreya & Burton, 2007), as well as the weak negative correlations that can be observed sometimes when matching accuracy is considered at the observer level (e.g., Kokje et al., 2018; Megreya & Burton, 2007).

The question of main interest concerned how this match–mismatch dissociation can be reconciled with visual similarity as the potential basis of face-matching decisions. For match trials, similarity judgements of faces correlated positively with classification accuracy, whereby face pairings that received higher similarity ratings were more likely to be classified correctly, while those that received low similarity ratings generated a greater number of errors. Correspondingly, similarity correlated negatively with accuracy on mismatch trials, whereby the smaller the resemblance between two faces, the more likely they were to be classified as different identities. In addition, identification *errors* were also marked by a systematic difference in similarity judgements. Incorrectly classified matches, for example, received lower similarity ratings than when the same stimuli were classified correctly, and the opposite relationship was observed for mismatches. This is an intriguing result, as it suggests that face-matching errors arise from variation in how observers *perceive* the similarity of two faces.

Overall, this experiment provides multiple sources of evidence that similarity plays a fundamental role in how observers decide whether two unfamiliar faces show the same person or different people. In conjunction with the by-item accuracy correlations, however, these findings offer somewhat contradictory interpretations. The first of these, based on the accuracy data alone, suggests that match and mismatch trials engage some separable processes. On the contrary, the similarity correlations also imply a common basis for judging whether pairs of faces are identity matches or mismatches.

## Experiment 2

In Experiment 1, match and mismatch stimuli were based on the same set of identities to demonstrate dissociation in the classification of these stimuli at the item level. It is also possible, however, that this design amplifies accuracy–similarity relationships across matches and mismatches, because the same identities are employed for both face-pair types. Experiment 2 therefore adapts a design that is employed more commonly in this field, by drawing match and mismatch trials from separate identity pools (see, for example, Bate et al., 2018; Fysh & Bindemann, 2018; White et al., 2015). The question of main interest here is whether similarity produces corresponding associations with match and mismatch trials when these are based on distinct identities. As in Experiment 1, this should be evident from a positive correlation of accuracy and similarity for matches, and the inverse relationship for mismatches.

### Method

#### Participants

A total of 95 undergraduate students (77 females, 18 males) from the University of Kent, with a mean age of 21 years (*SD* = 6.32), participated in this experiment in exchange for course credit. None of the participants had taken part in Experiment 1.

#### Stimuli and procedure

The stimuli in this study were taken from the short version of the KFMT (Fysh & Bindemann, 2018). The same 20 mismatch items as in Experiment 1 were employed, but 20 new matches were used. In contrast to Experiment 1, these stimuli were combined such that none of the identities occur in more than one stimulus pair, irrespective of whether this is a match or a mismatch. For full details of the KFMT, see Fysh and Bindemann (2018).

This study was conducted online using *Qualtrics* software. First, the stimuli were presented in a random order, along with the question, “Do the above two faces depict the same person or different individuals?” Participants classified stimuli by clicking on the relevant “same” or “different” response. In a second block of trials, observers then viewed each stimulus pair again in a randomised order, and rated the similarity of each pair on a 7-point scale.

### Results

#### Accuracy

Percentage accuracy was calculated for match and mismatch trials on a by-subject basis. Consistent with the by-subject analysis of Experiment 1, these data show a weak negative correlation between match and mismatch accuracy, *r* = −.20, *p* = .054.

#### Accuracy–similarity correlations

**Figure 3.**
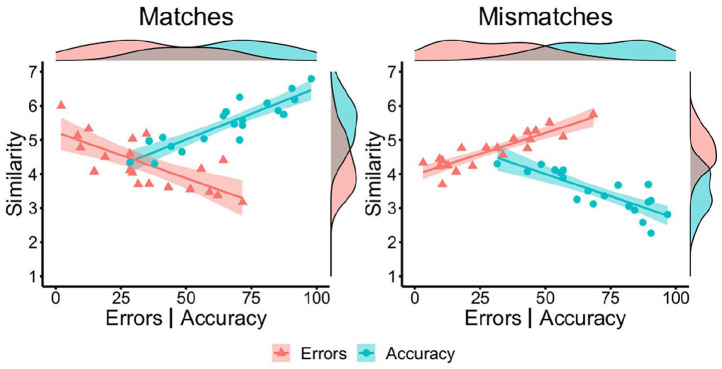
By-item correlations of accuracy (blue circles) and error percentage (red triangles) with similarity ratings for matches (left) and mismatches (right) in Experiment 2.

Next, we examined the similarity and accuracy of matches and mismatches at the item level. Mean similarity ratings for correctly classified match items (*M* = 5.48, *SD* = 0.70) were higher than for correctly classified mismatches (*M* = 3.47, *SD* = 0.59), *t*(38) = 9.81, *p* < .001, *d* = 3.10. As shown in Figure 3, a strong positive correlation was observed between accuracy and similarity for match items, *r* = .89, *p* < .001, and a strong negative relationship was found between accuracy and similarity for mismatch items, *r* = −.82, *p* < .001. A corresponding pattern was observed for incorrect trials, whereby mean incorrect similarity scores correlated negatively with error rates for match trials, *r* = −.73, *p* < .001, and positively with error rates for mismatches, *r* = .88, *p* < .001 (see Figure 3). Thus, match pairs were more likely to be classified correctly when their similarity was judged to be high, whereas accuracy was greatest for mismatches that were judged to be of low similarity.

#### Perceived similarity

As in Experiment 1, we next investigated whether the mean similarity ratings of correctly classified match and mismatch trials correlated with those of incorrectly classified trials. This revealed strong positive correlations for matches, *r* = .81, *p* < .001, and mismatches, *r* = .71, *p* < .001, which demonstrates that these ratings follow a similar pattern irrespective of whether correct or incorrect matching decisions are made. However, correctly classified match pairs received higher similarity ratings (*M* = 5.48, *SD* = 0.70) than when the same matches were classified incorrectly (*M* = 4.30, *SD* = 0.76), *t*(38) = 5.11 *p* < .001, *d* = 1.62. In turn, correctly classified mismatches received lower similarity ratings (*M* = 3.47, *SD* = 0.59) than their incorrectly classified counterparts (*M* = 4.71, *SD* = 0.52), *t*(38) = 7.04, *p* < .001, *d* = 2.23.

### Discussion

This experiment replicates the key aspects of Experiment 1, but with match and mismatch trials that are drawn from independent pools of identities. Despite this change, similarity correlated positively and strongly with accuracy for match items and negatively for mismatch items. Once more, this similarity link was also reflected in incorrect identifications, in two different ways. First, similarity ratings correlated positively for correctly and incorrectly classified face pairs, indicating that this information was extracted comparably by observers. At the same time, identification errors were marked by a shift in the similarity that observers perceived in faces, whereby matches were reported to be *less* similar, and mismatches to be *more* similar, when these were classified incorrectly. This indicates that the similarity of faces is systematically *mis*perceived when identification errors occur. Overall, these results converge by suggesting that both match and mismatch decisions are strongly guided by facial similarity.

The consistency of these similarity associations across Experiments 1 and 2 amplifies the surprising absence of a correlation in the classification accuracy of match and mismatch items. This absence of a positive match–mismatch association suggests that these stimuli are treated as separate challenges by the cognitive system and are resolved via different processes, despite being seemingly complementary aspects of the same task. On the contrary, it is difficult to reconcile this theoretical position with the finding that matches and mismatches relate to similarity strongly, consistently, and in a comparable manner. A possible explanation for these findings is that similarity is utilised differently to reach match and mismatch decisions. This is investigated in Experiment 3.

## Experiment 3

Experiments 1 and 2 demonstrate the theoretical paradox of face matching. Whereas classification of matches and mismatches is dissociable, these separable identification processes appear to be based on facial similarity information. One possibility to reconcile these characteristics of face matching is that similarity is utilised *differently* in match and mismatch decisions.

The accurate recognition of familiar faces is typically attributed to holistic processing mechanisms, by which faces are perceived as a single percept that can be processed at a glance (Maurer et al., 2002; Rezlescu et al., 2017; Richler & Gauthier, 2014). Such holistic processes appear to be less important for face matching. Face-matching performance improves, for example, when more viewing time is available, which indicates that a more analytical comparison of stimuli yields higher identification accuracy (Özbek & Bindemann, 2011). Observers are also good at matching computer-generated faces that vary by only one feature (Ramon & Rossion, 2010), or at recognising changes to individual features in newly learned schematic faces (Tanaka & Farah, 1993). Unfamiliar-face matching also correlates with object-matching tests that require identification of specific features (Burton et al., 2010; Megreya & Burton, 2006). There is therefore converging evidence that the perception of *features* is important for the identity processing of unfamiliar faces.

It is less clear which features carry the most relevant information for face matching. Open-mouth smiles, for example, appear to amplify discrepancies between identities (Mileva & Burton, 2018), while other studies suggest that the eyebrows (Megreya & Bindemann, 2018), nose (Rice et al., 2013), or ears, scars and blemishes (Towler et al., 2021, 2017) are important for face matching. This indicates that feature-based comparisons enhance face matching (Towler et al., 2019, 2021) but that the informativeness of specific face regions varies (e.g., Rice et al., 2013; Towler et al., 2017). In turn, this implies that different information towards a match or mismatch decision must be integrated across features. What is less clear is *how* information from different features is integrated in assessing the similarity of to-be-compared faces. In the current experiment, we therefore focus on the similarity information that is provided across different facial features to explore the relationship of similarity with the match-mismatch dissociation.

The basis of this experiment is that the ratings collected in Experiments 1 and 2 only allowed observers to make a single judgement to reflect the overall similarity of a face pair. This might be appropriate for match pairs for which, as these depict the same person, convergence in similarity ratings across features should be reasonably high. The faces in mismatches, however, typically bear some resemblance in appearance, while also depicting different people (see Burton et al., 2010; Fysh & Bindemann, 2018; Tummon et al., 2019). Thus, these face pairs might be more likely to vary in the facial similarity information that is provided across features. The key question that arises here is whether such systematic differences really do exist between matches and mismatches, and whether this can shed light on the differences in the processing of these types of face pairs. To investigate this, observers compared pairs of faces and then rated the similarity of their individual features. We then analysed how these similarity measurements relate to matching accuracy.

### Method

#### Participants, stimuli, and procedure

A total of 50 undergraduates from the University of Kent (8 males, 41 females, and one undisclosed), with a mean age of 20 years (*SD* = 2.35), participated for course credit. Stimuli and procedure were identical to Experiment 2, except that this experiment included a third block in which observers provided similarity judgements for the facial outlines, ears, eyes, noses, and mouths of each face pair stimulus. However, following the observation that the ears were not visible for all identities, similarity ratings for this feature were excluded from the final analysis. This experiment was conducted online using *Qualtrics* survey software.

### Results

#### Accuracy

Match and mismatch accuracies were calculated on a by-subject basis. These test subcomponents correlated negatively and weakly, *r* = −.32, *p* = .025.

#### Accuracy–similarity correlations

**Figure 4.**
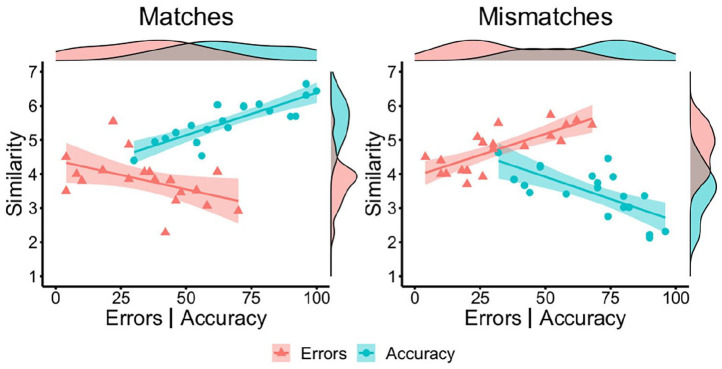
By-item correlations of accuracy (blue circles) and error percentage (red triangles) with similarity ratings for matches (left) and mismatches (right) in Experiment 3.

As in Experiments 1 and 2, mean similarity ratings for correctly classified match items (*M* = 5.57, *SD* = 0.61) were higher than for correctly classified mismatches (*M* = 3.46, *SD* = 0.72), *t*(38) = 9.98, *p* < .001, *d* = 3.16. Match similarity correlated positively with match accuracy, *r* = .85, *p* < .001, while mismatch similarity correlated negatively with mismatch accuracy, *r* = -.70, *p* < .001. Conversely, mean similarity scores correlated negatively with error rates for match trials, *r* = −.47, *p* = .042, and positively with error rates for mismatches, *r* = .77, *p* < .001. These correlations are illustrated in Figure 4.

**Figure 5.**
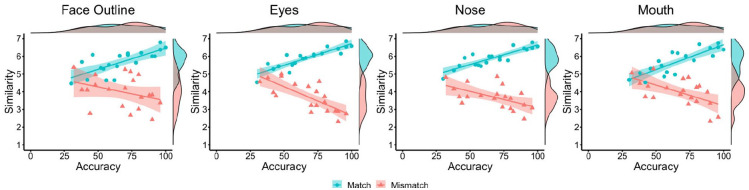
By-item correlations of accuracy and similarity ratings for the face outline (left), eyes (centre left), nose (centre right), and mouth (right) in Experiment 3, broken down by matches (blue circles) and mismatches (red triangles).

Next, the relationships between the similarity ratings for each facial feature and classification accuracy were investigated (see Figure 5). For match items, strong positive correlations were found between accuracy and the similarity of the facial outline, *r* = .74, *p* < .001, eyes, *r* = .89, *p* < .001, nose, *r* = .87, *p* < .001, and mouth, *r* = .84, *p* < .001. For mismatch items, accuracy did not correlate with the similarity of facial outline, *r* = −.34, *p* = .143, but was negatively associated with the similarity of the eyes, *r* = −.76, *p* < .001, nose, *r* = −.56, *p* = .011, and mouth, *r* = −.59, *p* = .006. Thus, similarity ratings for individual facial features generally follow the pattern observed for the whole face.

#### Independent feature similarity

**Figure 6.**
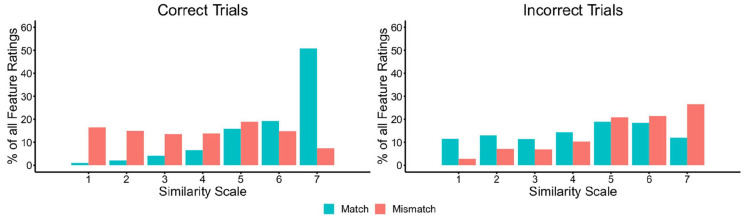
The percentage of feature judgements falling on each point of the similarity scale for matches and mismatches, broken down by correct (left) and incorrect trials (right) in Experiment 3.

To investigate differences between similarity ratings for facial features on match and mismatch trials, we next calculated the percentage of feature similarity ratings that fell on each point of the similarity scale. These data are provided in Figure 6, broken down for correctly and incorrectly classified trials.

Inspection of Figure 6 shows markedly different patterns for match and mismatch trials. For correct match trials, most features are given a high similarity rating of five, six, or seven. For correct mismatch trials, on the contrary, the responses for individual features are distributed more evenly across the scale. A reversal of this pattern is evident on trials that were classified incorrectly, whereby feature ratings for mismatches are more likely to fall on the high-similarity end of the scale, akin to correctly classified matches, whereas incorrect matches are distributed more evenly across the scale.

To analyse these observations systematically, the mean similarity ratings for each facial feature were calculated. A series of one-sample *t*-tests were then performed to compare the similarity of each facial feature to the mid-point (of 4) of the scale. For identity matches, this analysis confirmed that similarity scores were reliably greater than the scale mid-point for the face outline (*M* = 5.68, *SD* = 0.67), *t*(19) = 11.22, *p* < .001, *d* = 2.51, the eyes (*M* = 5.92, *SD* = 0.57), *t*(19) = 14.98, *p* < .001, *d* = 3.35, the nose (*M* = 5.92, *SD* = 0.53), *t*(19) = 16.29, *p* < .001, *d* = 3.64, and the mouth (*M* = 5.74, *SD* = 0.70), *t*(19) = 11.11, *p* < .001, *d* = 2.49. By contrast, the feature similarity ratings for the face outline (*M* = 4.00, *SD* = 0.90), eyes (*M* = 3.72, *SD* = 0.82), and mouth (*M* = 3.97, *SD* = 0.76) were comparable to the scale mid-point in mismatches, all *t*s ⩽ 1.52, all *p*s ⩾ .144, all *d*s ⩽ 0.34. Similarity ratings for the nose (*M* = 3.70, *SD* = 0.64) were also close to the scale mid-point, although this difference was approaching significance, *t*(19) = 2.09, *p* = .050, *d* = 0.47.

These analyses suggest that match and mismatch pairings are characterised differently in terms of feature similarity. On match trials, facial features typically fall on the higher end of the similarity scale, indicating that all features tend to convey similarity information that points to an “identity match” decision. For mismatch trials, on the contrary, the similarity ratings are more evenly distributed across the scale, suggesting ambiguity in the similarity information that the features of these face pairings convey.

#### Combined feature similarity

The analysis in Figure 6 presents the similarity ratings for the features of each face pair as independent events. To investigate the extent to which the features of the *same* items are consistently perceived to be similar, the mean similarity for each trial was calculated, based on the average rating of the facial outline, eyes, nose, and mouth features. The percentage of trials that received a particular mean rating along the 7-point scale was then analysed.

**Figure 7.**
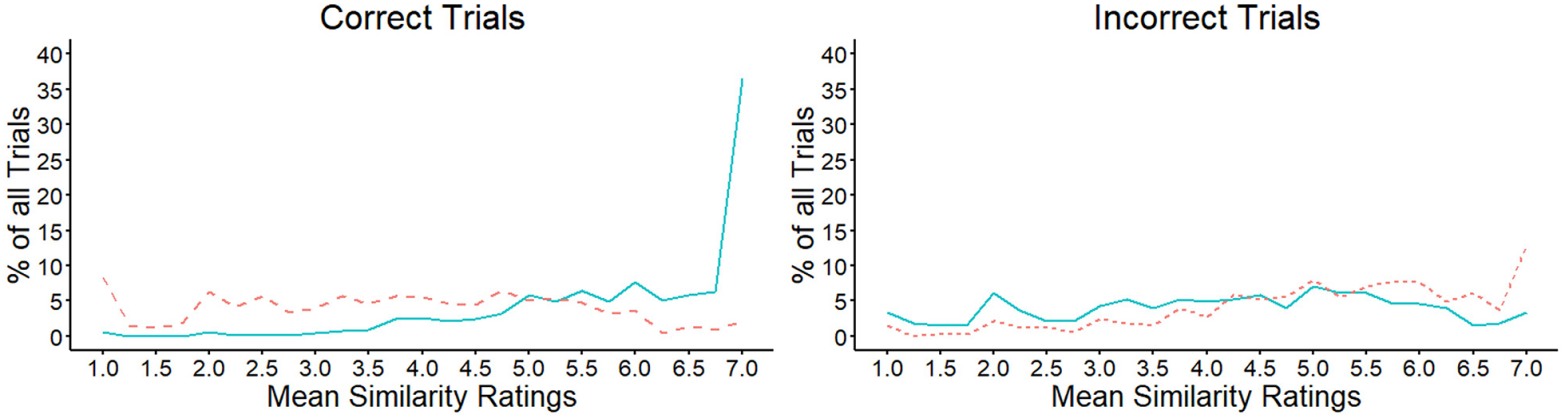
Mean similarity ratings for matches (solid line) and mismatches (dashed line) in Experiment 3, for correct (left) and incorrect (right) decisions.

These data are illustrated in Figure 7 and show that the mean similarity of facial features increased across the scale for correct match decisions. In line with these observations, a strong positive correlation was observed when the scale scores were correlated with the percentage of match trials that fell on each of the scale points, *r* = .62, *p* = .001. This indicates that this percentage increased as the *shared similarity* across features also increased. A different pattern was observed for correctly classified mismatches, for which the trial percentages were distributed more evenly across the similarity scale and did not correlate with the scale points, *r* = −.28, *p* = .177. Incorrect mismatches also showed a similar pattern to correct matches across the similarity scale, with the percentages that fall on each scale point increasing systematically towards the higher end of the scale, *r* = .84, *p* < .001. In turn, the pattern for incorrect matches appeared similar to that of correct mismatches, by showing a more even distribution of responses across all scale points, *r* = .26, *p* = .204.

Overall, these data indicate that similarity information across the different features of face pairs operates differently in matches and mismatches. In matches, this information typically converges towards the high end of the similarity scale, which indicates that the different facial features in these stimulus pairs tend to point to the same overall outcome. For mismatches, a corresponding convergence is not found, suggesting that the features of these face pairs provide much more varied similarity information.

#### Similarity convergence

The data in Figures 6 and 7 suggest that ratings for the different features within a face pair may exhibit a greater range in scores on mismatch than match trials. To confirm this observation, the standard deviation of the similarity ratings to all features in a face pair was calculated for each individual item. The means of these standard deviations were then also calculated on a by-item basis, as an index of the variability that similarity ratings exhibit across the features of match and mismatch pairs.

**Figure 8.**
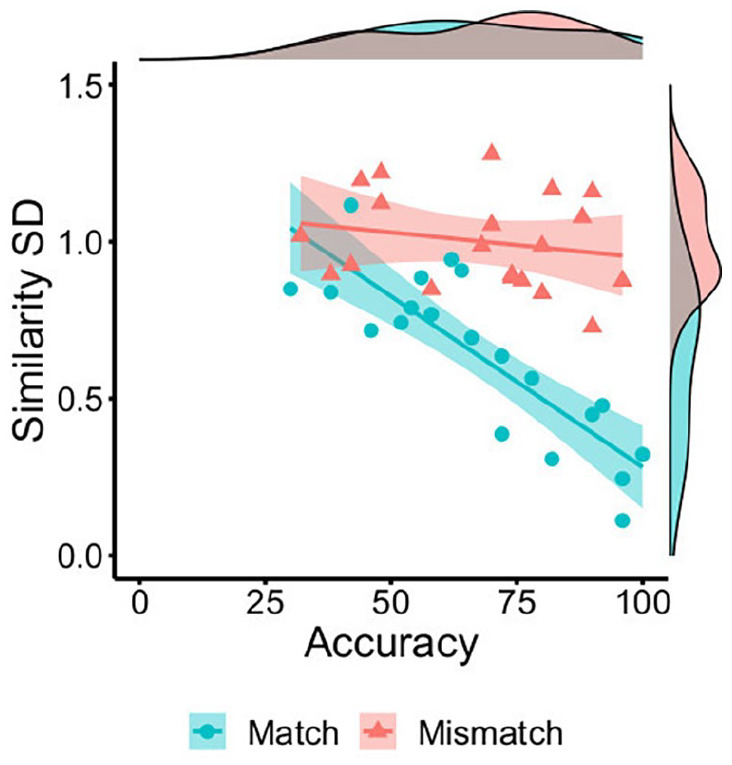
By-item correlations of accuracy percentage with the mean standard deviation of feature similarity ratings for matches (blue circles) and mismatches (red triangles) in Experiment 3.

An independent samples *t*-test confirmed that this variability was greater for mismatches (*M* = 1.00, *SD* = 0.15) than matches (*M* = 0.64, *SD* = 0.27), *t*(38) = 5.30, *p* < .001, *d* = 1.68. Moreover, this variability was negatively associated with accuracy for match trials, *r* = −.84, *p* < .001, but dissociated from accuracy on mismatch trials, *r* = −.21, *p* = .385 (see Figure 8). These data indicate that the convergence of feature ratings within face pairs is associated with higher identification accuracy for matches, whereas neither divergence (high *SD*) nor convergence (low *SD*) of facial feature similarity can unanimously account for mismatch judgements.

### Discussion

This experiment explored how similarity of individual facial features influences classification of match and mismatch pairs. We replicated the main findings of Experiments 1 and 2, by showing that the overall similarity of faces correlated positively with accuracy on match trials, and negatively on mismatch trials. Experiment 3 extends these findings by including similarity judgements for the face outline, eyes, nose, and mouth. These ratings showed that the similarity of individual facial features also correlated positively with accuracy for match items, akin to the similarity ratings for the whole face. Conversely, on mismatch trials the similarity of the eyes, nose, and mouth correlated negatively with identification accuracy.

Importantly, the similarity ratings for facial features generated additional data that provide insight into why a positive accuracy association for match and mismatch trials is not found. The similarity ratings for identity matches show that the individual features of these face pairs generally provide coherent information, by consistently pointing to the same identification outcome. For example, individual similarity ratings for all features were generally high (see Figures 5 and 6). And when similarity ratings were aggregated across features, the majority of match stimuli also received high scores (see Figure 7), indicating that the different features of these face pairs generally converged on the same identification outcome. This convergence was also evident when the range of similarity scores was considered for each face pair, based on the standard deviation of similarity judgements to all facial features (Figure 8).

A different picture emerged on mismatch trials, for which individual feature ratings and mean scores across the features of face pairs were distributed evenly across the full range of the similarity scale (see Figures 6 and 7). These differences in similarity were complemented by the standard deviation of feature ratings, which showed that variability in ratings across the features of a face pair was greater in mismatches than matches (see Figure 8). Together, these findings indicate that match and mismatch trials cannot be dichotomised based on overall similarity alone, as this information is generally expressed differently across the features of faces within these stimulus pairs.

## Experiment 4

Experiment 3 demonstrates that similarity is expressed differently across match and mismatch face pairs. In matches, similarity information from different facial features converges in pointing to an identification outcome, whereas this information is much more variable in mismatches, neither consistently converging nor diverging towards identification decisions. These findings were obtained with face pairs from the KFMT, which is designed to present a challenging face-matching test in which match trials portray identities across time intervals of several months (Fysh & Bindemann, 2018). This experiment examines whether these findings generalise to a matching test that is constructed to reflect different conditions. In the Glasgow Face Matching Test (GFMT), observers match pairs of faces that were taken under highly controlled conditions, and with match trials that constitute images of the same person that were acquired only a few minutes apart (Burton et al., 2010). In comparison with the KFMT, this provides optimised conditions that result in higher face-matching accuracy (see Fysh & Bindemann, 2018). The KFMT and GFMT therefore have different face-image characteristics, but both tests have been employed extensively in face-matching research. Here we investigate whether the similarity effects that were obtained with the KFMT in Experiment 3 also persist for face pairs from the GFMT.

### Method

#### Participants, stimuli, and procedure

A total of 51 participants (25 females, 26 males) with a mean age of 34 years (*SD* = 12.48) were recruited via Prolific Academic to complete this study in exchange for a small fee. The design and procedure were identical to Experiment 3, except that the stimuli were replaced with 40 face pairs (20 matches, 20 mismatches) from the GFMT. Each face-pair stimulus comprised of two high-quality digital face images measuring 350 pixels in width, which were taken under controlled lighting with a neutral expression and frontal pose. Both images in a face pair were taken with different image-capture devices and, in the case of identity matches, approximately 15 min apart (for more information, see Burton et al., 2010). The face images were positioned on the left and right of the screen centre on a white background and were presented at a resolution of 72-ppi. This experiment was conducted online using *Qualtrics* survey software.

### Results

#### Accuracy

All data were analysed following the same format as Experiment 3. First, percentage accuracy was calculated on a by-subject basis for match and mismatch trials. These data were not correlated, *r* = −.10, *p* = .482.

#### Accuracy–similarity correlations

**Figure 9.**
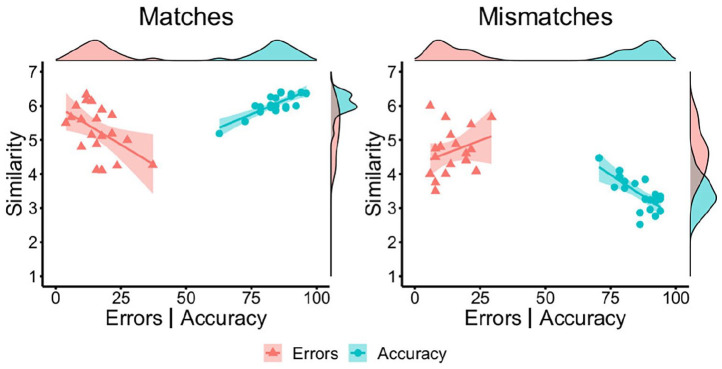
By-item correlations of accuracy (blue circles) and error percentage (red triangles) with similarity ratings for matches (left) and mismatches (right) in Experiment 4.

Mean similarity for match items (*M* = 6.05, *SD* = 0.31) was higher than for mismatches (*M* = 3.40, *SD* = 0.48), *t*(38) = 20.59, *p* < .001, *d* = 6.51. Match similarity correlated positively with match accuracy, *r* = .81, *p* < .001, and mismatch similarity correlated negatively with mismatch accuracy, *r* = −.72, *p* < .001. The inverse pattern was observed for incorrect match trials, where similarity correlated negatively with error rates, *r* = −.51, *p* = .023. For incorrect mismatches, the correlation of accuracy and similarity was not significant, *r* = .29, *p* = .211. These data are illustrated in Figure 9.

**Figure 10.**
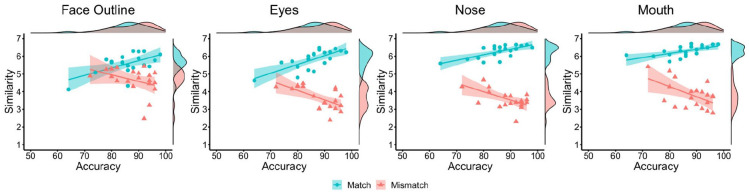
By-item correlations of accuracy and similarity ratings for the face outline (left), eyes (centre left), nose (centre right), and mouth (right) in Experiment 4, broken down by matches (blue circles) and mismatches (red triangles).

Further correlations were conducted to investigate the relationships between face-matching accuracy and feature-based similarity judgements (see Figure 10). For match items, positive correlations were found between accuracy and similarity ratings of the face outline, *r* = .57, *p* = .008, the eyes, *r* = .72, *p* < .001, nose, *r* = .69, *p* < .001, and mouth, *r* = .63, *p* = .003. For mismatch items, accuracy did not correlate with the similarity of face outline, *r* = −.37, *p* = .109, but was negatively associated with the similarity of the eyes, *r* = −.62, *p* = .004, nose, *r* = −.59, *p* = .006, and mouth, *r* = −.54, *p* = .014.

#### Independent feature similarity

**Figure 11.**
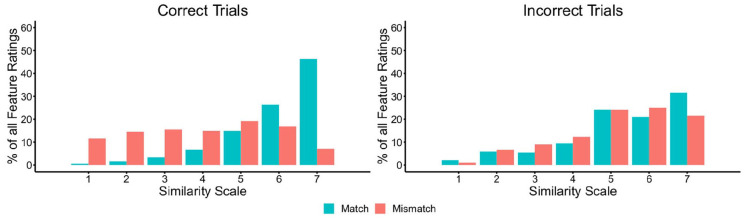
The percentage of feature judgements falling on each point of the similarity scale for matches and mismatches, broken down by correct (left) and incorrect trials (right) in Experiment 4.

As in Experiment 3, the percentage of features that were classified at each point of the similarity scale was calculated (see Figure 11). These data show that the majority of features were given high similarity ratings on correct match trials, whereas these ratings were distributed across the scale on correct mismatch trials. On incorrect trials, the feature ratings of both matches and mismatches generally fell on the higher end of the similarity scale.

To analyse these observations systematically, the mean similarity ratings for each facial feature were calculated and compared via one-sample *t*-tests to the scale mid-point (of 4). For identity matches, this analysis confirmed that similarity ratings were reliably greater than this mid-point for the face outline (*M* = 5.58, *SD* = 0.57), *t*(19) = 12.27, *p* < .001, *d* = 2.75, the eyes (*M* = 5.82, *SD* = 0.54), *t*(19) = 15.00, *p* < .001, *d* = 3.35, nose (*M* = 6.26, *SD* = 0.36), *t*(19) = 28.12, *p* < .001, *d* = 6.29, and mouth (*M* = 6.29, *SD* = 0.30), *t*(19) = 34.63, *p* < .001, *d* = 7.74. For mismatch items, the feature similarity ratings for the face outline (*M* = 4.64, *SD* = 0.72) were greater than the scale mid-point, *t*(19) = 4.02, *p* < .001, *d* = .90, whereas the ratings for the eyes (*M* = 3.63, *SD* = 0.61) and the nose (*M* = 3.61, *SD* = 0.57) were significantly below four, *t*(19) = 2.71, *p* = .014, *d* = .61 and *t*(19) = 3.10, *p* = .006, *d* = .69, respectively. Similarity ratings for the mouth (*M* = 3.81, *SD* = 0.75) were close to the scale mid-point, *t*(19) = 1.12, *p* = .276, *d* = .25.

#### Combined feature similarity

**Figure 12.**
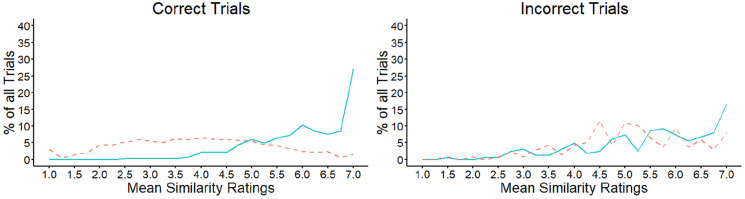
Mean similarity ratings for matches (solid line) and mismatches (dashed line) in Experiment 4, for correct (left) and incorrect (right) matching decisions.

We next calculated the mean similarity for each trial in the experiment, based on the average rating of the facial outline, eyes, nose, and mouth features, to investigate the extent to which the features of the *same* face pairs are *consistently* perceived to be similar. The percentage of trials that received a particular mean rating along the 7-point scale was then calculated. These data are illustrated in Figure 12 and show that the mean similarity of facial features increased across the scale for correct match decisions, *r* = .77, *p* < .001. For correctly classified mismatches, on the contrary, the trial percentages were distributed more evenly across the similarity scale and did not correlate with the scale points, *r* = −.12, *p* = .560. In addition, incorrect matches and mismatches showed a similar pattern to correct matches across the similarity scale, with the percentages of trials increasing systematically towards the higher end of the scale, *r* = .84, *p* < .001 and *r* = .69, *p* < .001, respectively.

#### Similarity convergence

**Figure 13.**
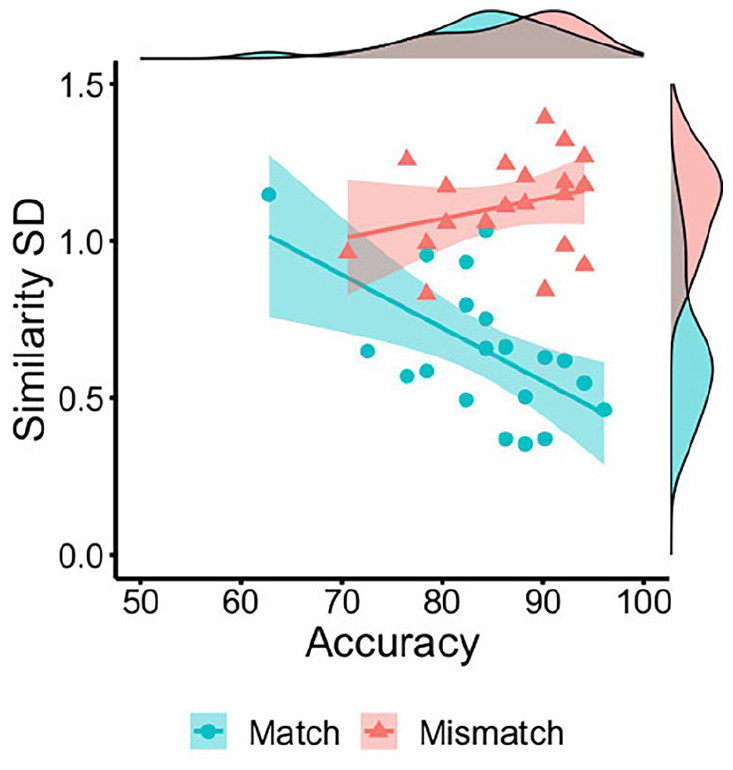
By-item correlations of accuracy percentage with the mean standard deviation of feature similarity ratings for matches (blue circles) and mismatches (red triangles) in Experiment 4.

In a final step, the standard deviation of similarity ratings to all features in a face pair was calculated for each item. The means of these standard deviations were then also calculated on a by-item basis, as an index of the variability that similarity ratings exhibit across features in match and mismatch pairs. An independent samples *t*-test confirmed that this variability was greater for mismatches (*M* = 1.11, *SD* = 0.16) than matches (*M* = 0.65, *SD* = 0.22), *t*(38) = 7.52, *p* < .001, *d* = 2.38. This variability was also negatively associated with accuracy for match trials, *r* = −.59, *p* = .006, but dissociated from accuracy on mismatch trials, *r* = .29, *p* = .219 (see Figure 13).

### Discussion

This experiment examined whether the similarity effects that were observed in Experiment 3 with face pairs from the KFMT can be replicated with the GFMT, which measures face-matching accuracy under highly optimised conditions (see Burton et al., 2010; Fysh & Bindemann, 2018). The results were remarkably similar to Experiment 3, with all key findings replicated closely. Accuracy correlated strongly with similarity, but an in-depth analysis of similarity judgements showed that these characterised match and mismatch face pairs differently. In matches, the individual features of paired faces were generally perceived to be similar. In mismatches, on the contrary, features within face pairs were more variable in the similarity information that they conveyed, both within and across mismatch face pairs (see Figures 12 and 13). These results confirm that similarity of facial features operates differently across the two trial types, typically converging to point to an identity match verdict when two photos of the same person are shown but providing a mixture of similar and dissimilar identity information when two different identities are depicted.

## General discussion

Substantial research is now available on unfamiliar-face matching, but an understanding of *how* this task is accomplished is still limited. This knowledge gap is emphasised by a dissociation in the classification of identity matches and mismatches, which suggests that these seemingly obverse aspects of the same task are driven by different cognitive processes (Bate et al., 2018; Kokje et al., 2018; Megreya & Burton, 2007; Megreya et al., 2011). The paradoxical nature of this dissociation becomes clear when one considers that this task is constrained by the amount of visual information within a given image (see Jenkins & Burton, 2011). Thus, the visual similarity of to-be-compared face images should form the basis of both match and mismatch decisions, but how this relates to the dissociation in the classification of these different face pairings is unclear. To understand the relationship of match and mismatch trials, our observers completed the KFMT (Fysh & Bindemann, 2018) and GFMT (Burton et al., 2010) and rated the similarity of the face pairs in these tests.

Across four experiments, we observed consistently strong similarity effects, whereby this correlated positively with accuracy for matches and negatively for mismatches, both for pairs of whole faces and of individual features. This relationship was also evident from error trials, as matches were reported to be less similar, and mismatches to be more similar, when these were classified incorrectly. These correlations converge with other work in suggesting that similarity judgements provide a reliable route to face matching (White et al., 2013), and that important information for facilitating this identification process is carried by individual facial features (e.g., Abudarham et al., 2019; Abudarham & Yovel, 2016; Megreya & Bindemann, 2018; Towler et al., 2017). However, these general similarity effects provide no direct insight into why accuracy for match and mismatch trials fails to correlate positively.

A more in-depth analysis of individual facial features in Experiments 3 and 4 revealed a clearer picture. For match pairs, similarity of individual facial features was generally rated highly. And when these similarity ratings were aggregated across the features of a face pair, the majority of match trials produced scores that were at or near ceiling. This demonstrates that similarity was shared across features of faces belonging to the same person, and that this information therefore converged to point to the same identification outcome. A different pattern emerged on mismatch trials, whereby individual facial features elicited a much greater variety of similarity responses. Aggregated across the features of a face pair, these similarity ratings neither consistently converged nor diverged, indicating that the constituent face regions of mismatches can vary substantially in terms of the identity information they provide.

These different similarity profiles indicate that distinct processes are required to classify identity matches and mismatches. Because the similarity of facial features converges in match trials, we propose that information across features can be *accumulated* to reach an identification decision, perhaps akin to how information is accumulated through sequential sampling in diffusion models (e.g., Forstmann et al., 2016; Ratcliff & Smith, 2004). Our findings indicate also that sameness is not an unequivocal indicator of shared identity, as high similarity of individual features can be observed *both* in matches and mismatches. Again, this supports the idea that identity match decisions must be substantiated by accumulating evidence across face regions, with one purpose of this process being to ascertain the absence of dissimilarity (i.e., to ensure that no meaningful differences between faces in a pair are found). In identity mismatches, on the contrary, the role of similarity is more complex due to the fact that these face pairs contain a combination of similar and dissimilar features. When confronted with this situation, mismatches cannot be identified simply by accumulating convergent information. Under these circumstances, images of different identities must therefore be distinguished via a cognitive process that can *evaluate* the available evidence, to compare whether similarities or dissimilarities carry more meaning.

This theoretical framework provides parsimony between two inconsistent findings—the absence of an association between the classification of matching and mismatching face pairings, yet the strong accuracy–similarity correlations that are observed with both types of stimuli. We suggest that accuracy for match and mismatch trials fails to correlate positively because different processes underpin the classification of these face pairings, based either on the *accumulation* of convergent similarity information from different aspects of faces or, when this fails, the *evaluation* of divergent information. Therefore, while the role of similarity as the basis for face-matching decisions appears incompatible with the match–mismatch dissociation at first sight, we suggest that similarity can reconcile this dissociation when the different processes underlying the classification of match and mismatch face pairings are understood.

A key aspect of this theory is that dissimilarities generally occur less frequently than similarities (see Figures 6 and 11) and therefore carry more weight in reaching an identification decision in face matching. Even different people may display some similarities in specific facial features, and so evaluating the identity relevance of such occurrences is difficult when this information is considered in isolation. The presence of dissimilarities, on the contrary, can provide direct support that a face pairing comprises of two different people. A shape match between the noses of two faces, for example, is unlikely to be indicative of an identity match on its own considering that many different people may have noses that are similar in appearance, whereas the probability that two faces with very different shaped noses belong to the same person is low. However, if accumulation and evaluation processes are applied to face matching, then they are also unlikely to divide match and mismatch identification completely. Our data indicate also, for example, that some matches provide ambiguous similarity information, whereas the features of some mismatching face pairs also converge on a mismatch outcome (see Figures 7 and 12). Thus, it appears that match and mismatch classifications are *generally*—but not exclusively—derived from different processes.

What are the potential neural mechanisms through which these perceptual match and mismatch decisions might be performed? In the study of how newly learned faces are subsequently recognized, isolable components have been identified that correspond with the correct decision that a face has been seen before (i.e., an “old” face) and the correct rejection of faces that had not been learned (a “new” face). These isolable components appear to be linked to dissociable deficits in different brain regions. The correct recognition of learned faces appears to be impaired by damage to brain regions in the right temporal lobe (e.g., Damasio et al., 1990; De Renzi et al., 1994; Sergent & Signoret, 1992), whereas right frontal lobe damage has been linked to the failure to reject a new face as unknown (Rapcsak et al., 2001, 1999). On the basis of these findings, a model has been posited whereby temporal brain regions signal resemblance between a previously learned face and a target (i.e., an identity match). However, as the faces of different people can bear similarities in appearance, this temporal lobe component is complemented with a frontal “executive” mechanism to regulate the correct rejection of new faces, by evaluating similarities and differences between identities (Rapcsak et al., 1999).

In support of this model, subsequent research applying principal component analysis has shown that identifications of old faces and rejections of new faces are driven by separable processing components in neurologically healthy participants (Bartlett et al., 2009). This research also included conjunction stimuli, which were created by combining features of two different face identities (e.g., the eyes, nose, and mouth of face A, with the hair and face outline of face B) that had been learned. Notably, the false recognition of these conjunction faces was linked to the same principal component as correct identifications, indicating a process that codes the *amount* of resemblance between a study and a test face. These findings fit with a model in which the temporal lobe computes the resemblance between two faces, whereas a frontal executive component reduces the false decisions that two faces are the same, by checking the associative information between the various parts of a face that are encountered together (Bartlett et al., 2009). This model resonates with the framework that we have put forward here to understand face matching. Both lines of research concern the identification of unfamiliar (or newly learned/previously unfamiliar) faces and demonstrate a dissociation between hits (i.e., accepting an “old” face or an identity match) and correct rejections (i.e., rejecting a “new” face or a mismatch). These lines of research converge also on the basis that old/match decisions are based on the *accumulated* resemblance between two faces, whereas new/mismatch decisions require a decision-making mechanism that can *evaluate* shared similarities and differences between two faces to determine when two facial identities do *not* match.

## Future directions

While a theoretical account in which match–mismatch decisions are based on information accumulation (matches) and evaluation (mismatches) provides the best fit for the current data, a number of questions arise from this proposal. For example, it is tempting to characterise the accumulation of feature information in terms of holistic processing, whereby the component features of faces are combined to form a single percept (Maurer et al., 2002; Rezlescu et al., 2017; Richler & Gauthier, 2014). We note, however, that viewing conditions favouring holistic over featural processing, such as the short peripheral presentation of faces, lead to an increase in mismatch rather than match decisions (Özbek & Bindemann, 2011). Moreover, there is already substantial evidence that the perception of individual features, rather than holistic processing, is particularly important for face matching (e.g., Burton et al., 2010; Megreya & Burton, 2006; Ramon & Rossion, 2010; Towler et al., 2019). Indeed, we only examined a subset of features, which is unlikely to account fully for how observers compare faces. Evidence suggests, for example, that observers also use information from the eyebrows (Megreya & Bindemann, 2018), jawline (Towler et al., 2017), and skin tone (Rice et al., 2013) when matching faces.

Consideration of the features that are used for matching decisions also raises the question of whether the match–mismatch dissociation can be linked to how faces are searched for information. It is possible, for example, that classification of matches might entail an exhaustive search to accumulate information, whereas information search in mismatch pairs can be terminated early, following the detection of a highly individuating difference between faces (e.g., scars or blemishes; see Moreton, 2021; Towler et al., 2017). Response time and eye movement data indicate that matches and mismatches cannot be distinguished easily along these lines (see, for example, Fysh & Bindemann, 2018; Özbek & Bindemann, 2010). This could reflect that detection of a distinguishing similarity feature in a mismatch (or match) might require exhaustive search until this is found or that it could be detected after only partial search. Thus, while search for information must be an important component of face matching, it seems unlikely that a simple distinction between partial and exhaustive search can explain broader differences in the identification of matches and mismatches.

While the current study demonstrates a clear link between similarity judgements and matching accuracy, we also note that similarity remains a difficult concept to define (see, for example, Algom & Fitousi, 2016). In the current experiments, similarity ratings varied for the correct and incorrect classification of the same face pairs. The faces in identity match pairs, for example, were rated as less similar when these were classified incorrectly as mismatches compared with when correct identifications were made. This indicates that there are differences between observers in the *perception* of facial similarity, and that these differences can be linked directly to the identification decisions that are reached. These findings might reflect that face-matching decisions require observers to have some similarity criteria for stimuli that inherently also contain dissimilarities. Even identity matches, for example, are based on *different* images of the same person. The challenge of face matching is therefore in determining whether differences between two face images reflect within-person variability (i.e., dissimilarities) in appearance or between-person similarities (see Bindemann & Burton, 2021), and the established difficulty of the task demonstrates that this combination can be difficult to resolve.

The mixture of within-person variability and between-person similarity is likely to depend on the stimuli at hand. In face-matching tests such as the GFMT and KFMT, for example, the different similarity profiles for matches and mismatches might arise in part because of how mismatches are constructed for face-matching research, by pairing two different identities that could conceivably be the same person (e.g., Bindemann et al., 2021; Burton et al., 2010; Fysh & Bindemann, 2018). This approach approximates the challenges that are associated with matching faces in real-world settings such as passport control, in which impersonation attempts can be rather compelling, but it also means that mismatched stimuli are specifically designed to be mistaken for identity matches. The contrary cannot be stated for identity matches, for which two images are paired on the single basis that they depict the same person, irrespective of similarity. It therefore makes sense to evaluate ambiguous or contradictory evidence to resolve mismatch pairings, given that these trials simultaneously incorporate similarity by virtue of how they are constructed but also dissimilarity because different people are depicted. One might anticipate that under different circumstances, for example, where mismatches might be paired based on broad categorical differences such as sex or race, a positive correlation might be found between match and mismatch accuracy on the basis that observers could resolve both types of trials via a single, shared decision process. However, the study of such broad distinctions is likely to contribute little to our understanding of person identification.

## Notes

1. The data for all experiments reported here can be accessed at https://osf.io/epcvz
